# Association between common early-childhood infection and subsequent depressive symptoms and psychotic experiences in adolescence: a population-based longitudinal birth cohort study

**DOI:** 10.1017/S0033291720004080

**Published:** 2022-08

**Authors:** Anna B. Chaplin, Peter B. Jones, Golam M. Khandaker

**Affiliations:** 1 Department of Psychiatry, University of Cambridge, Cambridge, UK; 2 Cambridgeshire and Peterborough NHS Foundation Trust, Cambridge, UK

**Keywords:** ALSPAC, childhood infection, cohort study, depression, depressive symptoms, inflammation, psychosis, psychotic experiences

## Abstract

**Background:**

Childhood infections are associated with adult psychosis and depression, but studies of psychotic experiences (PEs) and depressive symptoms in childhood, adolescence, and early-adulthood are scarce. Previous studies have typically examined severe infections, but studies of common infections are also scarce.

**Methods:**

Using data from the Avon Longitudinal Study of Parents and Children (ALSPAC) birth cohort, we examined associations of the number of infections in childhood from age 1.5 to 7.5 years with depressive symptom scores at age 10, 13, 14, 17, 18, and 19 years, and with PEs at 12 and 18 years. We performed additional analysis using infection burden (‘low’ = 0–4 infections, ‘medium’ = 5–6, ‘high’ = 7–9, or ‘very high’ = 10–22 infections) as the exposure.

**Results:**

The risk set comprised 11 786 individuals with childhood infection data. Number of childhood infections was associated with depressive symptoms from age 10 (adjusted beta = 0.14; standard error (s.e.) = 0.04; *p* = <0.01) to 17 years (adjusted beta = 0.17; s.e. = 0.08; *p* = 0.04), and with PEs at age 12 (suspected/definite PEs: adjusted odds ratio (OR) = 1.18; 95% confidence interval (CI) = 1.09–1.27). These effect sizes were larger when the exposure was defined as very high infection burden (depressive symptoms age 17: adjusted beta = 0.79; s.e. = 0.29; *p* = 0.01; suspected/definite PEs at age 12: adjusted OR = 1.60; 95% CI = 1.25–2.05). Childhood infections were not associated with depressive/psychotic outcomes at age 18 or 19.

**Conclusions:**

Common early**-**childhood infections are associated with depressive symptoms up to mid-adolescence and with PEs subsequently in childhood, but not with these outcomes in early-adulthood. These findings require replication including larger samples with outcomes in adulthood.

## Introduction

Early-life infections are associated with an increased risk of serious mental health disorders in adulthood. There is an extensive literature linking prenatal maternal and childhood infections with schizophrenia and related psychotic disorders, which are associated with both central nervous system (CNS) and non-CNS infections (Benros et al., 2011; Brown & Derkits, 2010; Dalman et al., 2008; Khandaker et al., 2018; Khandaker, Zimbron, Dalman, Lewis, & Jones, 2012; Khandaker, Zimbron, Lewis, & Jones, 2013). Early-life exposure to infection may result in activation of an acute inflammatory response potentially affecting neurodevelopment (Boulanger & Shatz, 2004). Acute inflammation causes reductions in neuroplasticity, an important process for brain functioning, which may lead to neuropsychiatric outcomes later on (De Picker, Morrens, Chance, & Boche, 2017). Compared with psychosis, longitudinal studies of early-life infection and depression are relatively rare, though inflammation is increasingly thought to play a role in the pathogenesis of depression (Dantzer, O'Connor, Freund, Johnson, & Kelley, 2008; Khandaker, Dantzer, & Jones, 2017; Miller, Maletic, & Raison, 2009). Most of the existing longitudinal studies of early-life infection have focused on psychosis and depression in adults (Bechter et al., 2010; Benros et al., 2011; Eisenberger et al., 2010; Harrison et al., 2009; Laske et al., 2008; Meyer, Schwarz, & Müller, 2011). Longitudinal studies of depressive and psychotic experiences (PEs) during childhood/adolescence are scarce (Goodwin, 2011). Childhood/adolescent depressive symptoms are associated with adult depression (Copeland, Shanahan, Costello, & Angold, 2009; Dunn & Goodyer, 2006; Fombonne, Wostear, Cooper, Harrington, & Rutter, 2001; Lewinsohn, Rohde, Seeley, Klein, & Gotlib, 2000). PEs during childhood/early-adolescence may be part of typical development and are transient for most individuals. However, population-based longitudinal studies suggest association between early-life PEs with the risk of psychotic disorders subsequently in adulthood (Poulton et al., 2000; Zammit et al., 2013), and with risk factors for schizophrenia including impaired neurodevelopment (Cannon et al., 2002; Kelleher & Cannon, 2011). PEs are also associated with adolescent psychiatric multi-morbidity (Kelleher et al., 2012), and with other psychiatric disorders in adulthood (Fisher et al., 2013). Therefore, studying adolescent PEs and depressive symptoms may offer insights into the development of adult psychotic and mood disorders.

Some previous longitudinal studies have considered the issue of timing, i.e. whether early-life infections are associated with psychotic outcomes closer to the time of exposure or subsequently after several years (Benros et al., 2011; Nielsen, Benros, & Mortensen, 2014), but such studies of depression are scarce. Moreover, previous studies have typically examined effects of severe infections (Benros et al., 2011, 2013), but studies of common childhood infections and subsequent depressive/psychotic outcomes, or studies of the number of childhood infection and subsequent depression are scarce. Prospective cohort studies with repeated measures of depressive symptoms and PEs over a long period are required to address these issues, but such studies are relatively rare.

Infections are common during childhood, but some children are disproportionately prone to high infection burden possibly due to genetic and environmental factors. While childhood infection severity, duration, and related hospitalization have been linked with depression and psychosis in adulthood (Benros et al., 2011, 2013; Nielsen et al., 2014), it is unclear whether the degree of childhood infection burden is associated with psychotic/depressive outcomes in childhood/adolescence. A high number of childhood infections may be a risk factor for psychiatric disorders, as a dose-response association between increasing number of childhood infection and adult mood or psychotic disorders has been reported (Benros et al., 2011, 2013). Early-life infections could also be a marker for shared genetic and environmental risk factors for infection and major psychiatric disorders (Nielsen, Laursen, & Mortensen, 2013). It is possible that inflammatory immune response during a critical developmental window is detrimental for brain development/function (Cordeiro, Tsimis, & Burd, 2015). A high burden of common childhood infections may also reflect underlying familial factors predisposing to infection, such as socioeconomic status, living conditions, and genetic factors (Nielsen et al., 2013). Therefore, studies investigating the effects of infection burden are required.

Using data from the Avon Longitudinal Study of Parents and Children (ALSPAC) (Boyd et al., 2013), a general population-representative prospective British birth cohort, we have investigated the longitudinal associations of childhood infections from age 1.5 to 7.5 years old with depressive symptoms measured six times from age 10 to 19 years and with PEs at age 12 and 18 years. We examined not only the number of childhood infections as exposure, but also the effect of infection burden, grouped as low, medium, high or very high. We hypothesized that a higher overall number of infections and very high infection burden would be associated with higher risks for depressive symptoms and PEs subsequently up to early-adulthood.

## Methods

### Description of cohort and sample

ALSPAC is a general population-based birth cohort in the former Avon County in the South West region of England. Initially, 14 541 pregnant women resident in the study catchment areas and with expected delivery dates between 1 April 1991 and 31 December 1992 were recruited into the cohort. Detailed information about the ALSPAC cohort can be found on the study website (http://www.bristol.ac.uk/alspac), and the sample characteristics and methodology have been previously described (Boyd et al., 2013; Fraser et al., 2013). For information on all available ALSPAC data, a fully searchable data dictionary is also available (http://www.bris.ac.uk/alspac/researchers/our-data).

The risk set for this study comprised 11 786 individuals with data on infections during childhood. Out of the risk set, the number of individuals with depressive symptoms data decreased from 6685 at age 10 to 3101 at age 19 (online Supplementary Fig. 1). From the risk set, PEs data were available for 6176 individuals at age 12 and for 4253 individuals at age 18 (online Supplementary Fig. 2). These samples formed the basis for the analyses presented.

Ethical approval for the study was obtained from the ALSPAC Ethics and Law Committee and the Local Research Ethics Committees. Written informed consent was provided by all participants. No financial compensation was given.

### Exposure measures

#### Childhood infections from age 1.5 to age 7.5 years

Approximately once per year when the child was aged between 1.5 and 7.5 years old, caregivers completed seven postal questionnaires about common childhood infections experienced by their child. These included German measles, measles, chickenpox, mumps, meningitis, cold sores, whooping cough, urinary infections, eye infections, ear infections, chest infections, tonsillitis/laryngitis, scarlet fever, influenza, cold and ‘other’.

Overall, 11 786 children had fully/partly completed infection questionnaires from the seven time-points. In the questionnaire about childhood infections, caregivers could tick the ‘Yes’ or ‘No’ boxes for infections that their child has had. Some people simply ticked the ‘Yes’ box to indicate that their child has had certain infections, but did not tick the ‘No’ box for infections their child did not have. In those instances, we coded the un-ticked boxes as ‘No’ that year rather than dropping the subject from analysis for missing data. However, if a parent had all boxes unticked, they were coded as missing, as we did not have any indication whether their child had any infection that year.

Childhood infection count was used as a standardized continuous variable. Infection was also used as a categorical variable representing the degree of infection burden: low (50th percentile and below; 0–4 infections), medium (51–75th percentile; 5–6 infections), high (76–90th percentile; 7–9 infections), and very high burden (above 90th percentile; 10–22 infections). These categories were based on a prior study and were chosen to capture the positive skew of childhood infection distribution (Mackinnon, Zammit, Lewis, Jones, & Khandaker, 2018).

### Outcome measures

#### Depressive symptoms at age 10, 13, 14, 17, 18, and 19 years

Depressive symptoms were self-reported by the child/young person using the Short Mood and Feelings Questionnaire (SMFQ) (Sharp, Goodyer, & Croudace, 2006). Depressive symptoms were measured at age 10 (mean age in years = 10.6; standard deviation (s.d.) = 0.3), 13 (mean = 12.8; s.d. = 0.2), 14 (mean = 13.8; s.d. = 0.2), 17 (mean = 16.7; s.d. = 0.2), 18 (mean = 17.8; s.d. = 0.4), and 19 years (mean = 18.6; s.d. = 0.5). The SMFQ is a widely used, age-appropriate, and validated tool comprising 13 items that cover core symptoms of depression and anxiety experienced in the past 2 weeks. Each item is scored zero (not true), one (sometimes true) or two (true) giving a total score of 0–26. Depressive symptoms were used as a continuous variable.

#### PEs at age 12 and 18 years

PEs were identified using the face-to-face, semi-structured Psychosis-Like Symptom Interview (PLIKSi) (Horwood et al., 2008; Zammit et al., 2013) conducted by trained psychology graduates in assessment clinics. PEs were coded according to the definitions and rating rules for the Schedules for Clinical Assessment in Neuropsychiatry, Version 2.0. (World Health Organisation, 1994). PLIKSi at age 12 has ‘fair’ inter-rater (kappa = 0.75) and test-retest (kappa = 0.48) reliability (Horwood et al., 2008). PLIKSi at age 18 has ‘good’ inter-rater (kappa = 0.83) and test-retest (kappa = 0.76) reliability (Zammit et al., 2013).

PLIKSi covers the three main domains of positive PEs: hallucinations (visual and auditory), delusions (spied on, persecuted, thoughts read, reference, control, grandiosity, and other), and thought interference (insertion, withdrawal, and broadcasting). After cross-questioning, interviewers rated PEs as ‘not present’, ‘suspected’, or ‘definitely present’. Uncertain responses were always ‘rated down’, and symptoms were rated as definite only if a clear example could be provided. For suspected or definite PEs, interviewers also recorded the frequency, affect, effects on social/educational/occupational function, help-seeking, and attributions including fever, hypnopompic/hypnogogic state, or illicit drugs.

PEs measured at age 12 refer to experiences from the previous 6 months while PEs measured at age 18 refer to experiences since age 12. In line with previous papers originally describing PEs in the ALSPAC birth cohort, we used three increasingly strict definitions for this outcome, i.e. any PEs (suspected or definite PEs); definite PEs; and definite PEs without attribution. The number of participants meeting each of the outcome definitions are different (online Supplementary Fig. 2) and the risk of each outcome was analysed separately. The comparison group for each outcome included all the individuals who did not meet that specific definition for the outcome (Khandaker, Zammit, Lewis, & Jones, 2014).

### Confounders

Based on previous studies, we included sex, ethnicity, birth weight, maternal social status, and parental history of severe depression or schizophrenia as these are associated with exposure and/or outcome and so could be confounders (Abel et al., 2010; Benros et al., 2013; Khandaker et al., 2012; Khandaker, Stochl, Zammit, Lewis, & Jones, 2014). Sex (binary) and birth weight (grams, continuous) were assessed at birth. Ethnicity was recorded as White, Black African, Black Caribbean, Black Other, Bangladeshi, Chinese, Indian, Pakistani, and Other. We re-coded this variable as White and Other due to low counts for non-White ethnic groups. Maternal social status was originally documented using Office of National Statistics categories (Office for National Statistics, 2016) and re-coded as non-manual (I, II and IIIa) and manual (IIIb, IV and V). The armed forces were excluded as the sample size for this category was too small (*n* = 4). Mothers and their partners completed separate questionnaires at 12 weeks gestation in which they self-reported having severe depression or schizophrenia, which were used as binary variables. See Supplementary Material for how these binary variables were defined.

### Statistical analysis

All analyses were carried out using R version 3.6.1. Regression analyses were performed before and after adjusting for potential confounders. All main analyses were based on the dataset after imputation of missing values for confounders.

#### Imputation of missing confounder variables

Regression analyses testing associations between exposure and outcomes were conducted after imputation of missing data for confounders (ethnicity, maternal social status, birth weight, parental schizophrenia, and parental severe depression) to increase the sample size. Sex had no missing data. The percentage of missing values across the relevant confounder variables varied between 1.5% and 35.0% (online Supplementary Table 1). We used the *TestMCARNormality* function to test whether data were missing completely at random (MCAR) (Jamshidian, Jalal, & Jansen, 2014). The hypothesis of MCAR was rejected at the 0.05 significance level. Using the *missing_compare* function, each variable returned significance (*p* < 0.05) with at least one other variable, suggesting that the data met the missing at random (MAR) assumption.

We used multiple imputations using fully conditional Markov chain Monte Carlo method for the above confounder variables plus auxiliary variables that were indicators of missingness. Exposure and outcome variables were also included. The selected auxiliary variables included: housing/living conditions, parental education/employment status, financial difficulties, life events, and maternal characteristics (age, body mass index, marital status, anxiety/post-natal depression, and smoking status).

We used the R package *mice* version 3.0 to create and analyse the multiply imputed datasets (van Buuren & Groothuis-Oudshoorn, 2011). Since missing data were present in 19.9% of subjects, we used 20 imputations as recommended (White, Royston, & Wood, 2011). The parameters of interest were estimated in each dataset separately and combined using Rubin's rules.

#### Associations of childhood infection with depressive symptoms and PEs

Linear regression was used to examine the association between the number of childhood infections/infection burden and depressive symptoms. Low infection burden (0–4 infections) was used as the reference group where infection burden was used as the exposure. Regression models were adjusted for sex, birth weight, maternal social status, ethnicity, and parental history of severe depression. Beta estimates and standard error (s.e.) from regression models are presented.

Logistic regression was used to calculate odds ratios (OR) and 95% confidence intervals (CI) for PEs at age 12 or age 18 associated with the number of childhood infections/infection burden. The low burden of infection (0–4 infections) was used as the reference group for the latter. Regression models were adjusted for sex, birth weight, maternal social status, ethnicity, and parental history of schizophrenia.

For all analyses, we used the maximum available sample for each outcome measure to increase statistical power. Holm-Bonferroni *p* value correction was performed to correct for multiple testing (online Supplementary Table 2) (Holm, 1979).

#### Sensitivity analysis exploring impact of missing data

To explore the potential impact of missing data we repeated our main analyses based on the complete case-set, defined as participants with no missing data for exposure, outcome or confounder measures. We then carried out a number of comparisons between different samples. (1) We compared the risk set (i.e. participants with data on childhood infection) with the complete case-set for depressive symptoms (i.e. data on childhood infection, all confounders, and depressive symptoms at all follow-ups are available). We did the same comparison for PEs. (2) We compared the analytic sample for depressive symptoms at age 19 years (i.e. data on both childhood infection and depressive symptoms at age 19 available) with the missing sample (i.e. childhood infection data present but depressive symptoms data at age 19 missing). We performed the same comparison for PEs at age 18 separately.

## Results

### Baseline characteristics of the sample

The risk set (*N* = 11 786) was predominantly of white ethnicity (97.7%) (Table 1). The average number of childhood infections in the total sample was 4.6 (s.d. = 3.2), and the median was 4 (inter-quartile range = 2–6). The number of children with very high infection burden (⩾90th percentile; 10–22 infections) was 947 (8.0%).

**Table 1.** Characteristics of participants in the risk set (using maximum available sample and after imputation of confounders)

Characteristics	Total participants		Very high infection burden (⩾10 infections)		Lower infection burden (<10 infections)		Difference between groups (*t* test *p* value)
	Sample	Value	Sample	Value	Sample	Value	
Confounders							
Sex – *no. female* (*%*)	11 786	5710 (48.4)	947	483 (51.0)	10 839	5227 (48.2)	0.10
Ethnicity – *no. white* (*%*)	11 786	11 511 (97.7)	947	922 (97.4)	10 839	10 588 (97.7)	0.63
Maternal social status – *no. manual* (*%*)	11 786	2621 (22.2)	947	184 (19.4)	10 839	2437 (22.5)	0.04
Birth weight – *mean* [*standard deviation* (s.d.)]	11 786	3.4 (0.5)	947	3.4 (0.5)	10 839	3.4 (0.5)	0.39
Parental history of severe depression – *no.* (*%*)	11 786	1677 (14.2)	947	158 (16.7)	10 839	1519 (14.0)	0.07
Parental history of schizophrenia – *no.* (*%*)	11 786	40 (0.3)	947	3 (0.3)	10 839	37 (0.3)	0.91
Exposure: number of childhood infections							
*Mean* (s.d.)	11 786	4.6 (3.2)	947	11.6 (1.8)	10 839	4.0 (2.5)	–
*Median* (*interquartile range*)	11 786	4 (2-6)	947	11 (10–12)	10 839	4 (2-6)	
Outcome: depressive symptoms– *mean* (s.d.)							
Age 10	6685	4.0 (3.5)	697	4.4 (3.9)	5988	4.0 (3.5)	<0.01
Age 13	6087	3.9 (3.8)	661	4.6 (4.3)	5426	3.9 (3.8)	<0.01
Age 14	5504	4.9 (4.5)	604	5.5 (4.8)	4900	4.8 (4.4)	<0.01
Age 17	4645	5.9 (5.6)	506	6.6 (5.8)	4139	5.8 (5.6)	<0.01
Age 18	4086	6.6 (5.3)	448	6.9 (5.2)	3638	6.5 (5.3)	0.17
Age 19	3101	6.8 (5.9)	345	7.1 (6.1)	2756	6.7 (5.8)	0.30
Outcome: psychotic experiences (PEs) – *no.* (*%*)							
Suspected/definite PEs age 12	6176	844 (13.7)	668	117 (17.5)	5508	727 (13.2)	<0.01
Definite PEs age 12	6176	350 (5.7)	668	47 (7.0)	5508	303 (5.5)	0.14
Definite PEs without attribution age 12	6176	289 (4.7)	668	39 (5.8)	5508	250 (4.5)	0.17
Suspected/definite PEs age 18	4253	385 (9.1)	469	49 (10.4)	3784	336 (8.9)	0.29
Definite PEs age 18	4253	206 (4.8)	469	22 (4.7)	3784	184 (4.9)	0.87
Definite PEs without attribution age 18	4253	177 (4.2)	469	18 (3.8)	3784	159 (4.2)	0.70

There was a general increase in depressive symptoms between age 10 and 19 years; mean SMFQ score at age 10 was 4.0 (s.d. = 3.5), and was 6.8 (s.d. = 5.9) at age 19 (Table 1). The percentage of participants with definite PEs was similar at age 12 (5.7%) and at age 18 (4.8%).

### Association between number of childhood infections and depressive symptoms

Number of childhood infections from age 1.5 to age 7.5 years was associated with subsequent depressive symptoms at age 10 (*N* = 6685; beta = 0.14, s.e. = 0.04; *p* = <0.01), 13 (*N* = 6087; beta = 0.24; s.e. = 0.05; *p* = <0.001), and 14 years (*N* = 5504; beta = 0.23, s.e. = 0.06; *p* = <0.001) (Table 2). Evidence for these associations remained after adjusting for potential confounders. Childhood infections were not associated with depressive symptoms at age 17, 18, or 19 years.

**Table 2.** Beta estimate (s.e.) for the association between 1 s.d. increase in childhood infections and depressive symptoms from age 10 to 19 years

Age (years)	Sample size (no.)	% with depression (SMFQ ⩾8)	Unadjusted		Adjusted^a^	
			Beta (s.e.) for depressive symptoms	*p* value	Beta (s.e.) for depressive symptoms	*p* value
**10**	6685	14.5	0.14 (0.04)	<0.01	0.14 (0.04)	<0.01
**13**	6087	14.6	0.24 (0.05)	<0.001	0.22 (0.05)	<0.001
**14**	5504	22.2	0.23 (0.06)	<0.001	0.21 (0.06)	<0.001
**17**	4645	28.3	0.14 (0.09)	0.11	0.17 (0.08)	0.04
**18**	4086	34.3	0.11 (0.09)	0.22	0.11 (0.08)	0.19
**19**	3101	34.2	0.11 (0.11)	0.34	0.11 (0.11)	0.31

a Adjusted for sex, birth weight, maternal social status, ethnicity, and parental history of severe depression.

After correction for multiple testing, evidence remained for an association between number of childhood infections and depressive symptoms at age 10 (*p* = 0.02), 13 (*p* = <0.001) and 14 years (*p* = <0.01) (online Supplementary Table 2).

### Association between number of childhood infections and PEs

Number of childhood infections was associated with subsequent PEs at age 12 years (*N* = 6176), including suspected/definite PEs (OR = 1.17, 95% CI = 1.09–1.26), definite PEs (OR = 1.16, 95% CI = 1.04–1.29), and definite PEs without attribution (OR = 1.17, 95% CI = 1.04–1.31) (Table 3). Evidence for these associations remained after adjusting for potential confounders. The number of childhood infections was not associated with PEs at age 18 years.

**Table 3.** Odds ratio (95% CI) for the association between 1 s.d. increase in childhood infections and psychotic experiences (PE) at age 12 and age 18 years

Age (years)	Psychotic experiences (PEs)	Sample size (no.)	% with PEs	Odds ratio (95% CI) for PEs	
				Unadjusted	Adjusted^a^
**12**	Suspected/definite	6176	13.7	1.17 (1.09–1.26)	1.18 (1.09–1.27)
	Definite only		5.7	1.16 (1.04–1.29)	1.16 (1.05–1.29)
	Definite without attribution		4.7	1.17 (1.04–1.31)	1.18 (1.05–1.32)
**18**	Suspected/definite	4253	9.1	1.07 (0.97–1.19)	1.08 (0.97–1.20)
	Definite only		4.8	0.93 (0.81–1.08)	0.94 (0.82–1.10)
	Definite without attribution		4.2	0.88 (0.75–1.03)	0.89 (0.76–1.05)

a Adjusted for sex, birth weight, maternal social status, ethnicity, and parental history of schizophrenia.

After correction for multiple testing, evidence remained for an association between number of childhood infections and suspected/definite PEs at age 12 (*p* = <0.001), but not with definite PEs (*p* = 0.12) or definite PEs without attribution at age 12 years (*p* = 0.12) (online Supplementary Table 2).

### Association between childhood infection burden and depressive symptoms

Compared with low infection burden, very high infection burden was associated with depressive symptoms at age 10 (beta = 0.38; s.e. = 0.16; *p* = 0.01), 13 (beta = 0.76; s.e. = 0.17; *p* = <0.001), 14 (beta = 0.66; s.e. = 0.21; *p* = <0.01), and 17 years (beta = 0.68; s.e. = 0.30; *p* = 0.02) (Table 4). Evidence for these associations remained after adjusting for potential confounders. Very high infection burden was not associated with depressive symptoms at age 18 (beta = 0.33; s.e. = 0.29; *p* = 0.26) or 19 years (beta = 0.41; s.e. = 0.38; *p* = 0.27).

**Table 4.** Beta estimate (s.e.) for the association between childhood infection burden and depressive symptoms from age 10 to 19 years

Age (years)	Infection burden between age 1.5 and 7.5 years (no. of infections)	Sample size (no.)	% with depression (SMFQ ⩾8)	Unadjusted		Adjusted^a^	
				Beta (s.e.) for depressive symptoms	*p* value	Beta (s.e.) for depressive symptoms	*p* value
**10**	Low (0–4)	2869	14.6	0.00 (reference)		0.00 (reference)	
	Medium (5–6)	1703	12.3	−0.23 (0.11)	0.03	−0.20 (0.11)	0.05
	High (7–9)	1416	14.9	0.06 (0.12)	0.65	0.09 (0.12)	0.47
	Very high (10–22)	697	18.1	0.38 (0.16)	0.01	0.36 (0.15)	0.02
**13**	Low (0–4)	2572	13.5	0.00 (reference)		0.00 (reference)	
	Medium (5–6)	1538	13.8	−0.03 (0.12)	0.84	−0.02 (0.12)	0.86
	High (7–9)	1316	15.0	0.22 (0.14)	0.11	0.21 (0.14)	0.14
	Very high (10–22)	661	20.0	0.76 (0.17)	<0.001	0.72 (0.17)	<0.001
**14**	Low (0–4)	2328	21.4	0.00 (reference)		0.00 (reference)	
	Medium (5–6)	1383	21.0	−0.19 (0.15)	0.20	−0.15 (0.15)	0.31
	High (7–9)	1189	22.9	0.18 (0.17)	0.31	0.18 (0.17)	0.28
	Very high (10–22)	604	26.8	0.66 (0.21)	<0.01	0.64 (0.21)	<0.01
**17**	Low (0–4)	1917	27.6	0.00 (reference)		0.00 (reference)	
	Medium (5–6)	1184	28.8	−0.18 (0.21)	0.37	−0.04 (0.20)	0.85
	High (7–9)	1038	26.7	−0.30 (0.24)	0.20	−0.15 (0.23)	0.53
	Very high (10–22)	506	33.0	0.68 (0.30)	0.02	0.79 (0.29)	0.01
**18**	Low (0–4)	1708	33.8	0.00 (reference)		0.00 (reference)	
	Medium (5–6)	1053	33.6	−0.11 (0.20)	0.58	−0.01 (0.20)	0.95
	High (7–9)	877	34.7	0.10 (0.24)	0.67	0.22 (0.23)	0.34
	Very high (10–22)	448	37.5	0.33 (0.29)	0.26	0.34 (0.29)	0.24
**19**	Low (0–4)	1236	34.2	0.00 (reference)		0.00 (reference)	
	Medium (5–6)	829	32.6	−0.04 (0.26)	0.89	0.01 (0.26)	0.97
	High (7–9)	691	35.3	0.26 (0.30)	0.39	0.36 (0.30)	0.22
	Very high (10–22)	345	36.2	0.41 (0.38)	0.27	0.43 (0.37)	0.25

a Adjusted for sex, birth weight, maternal social status, ethnicity, and parental history of severe depression.

After correction for multiple testing, evidence remained for the association between very high infection burden and depressive symptoms at age 13 (*p* = <0.001) and 14 years (*p* = 0.02) (online Supplementary Table 2). Please see Table 4 for risk associated with other categories of infection burden.

### Association between childhood infection burden and PEs

Compared with low infection burden, very high infection burden was associated with suspected/definite PEs at age 12 years (OR = 1.59, 95% CI = 1.24–2.04). Evidence for this association remained after adjusting for potential confounders. Very high infection burden was not associated with PEs at age 18 (Table 5).

**Table 5.** Odds ratio (95% CI) for the association between childhood infection burden and psychotic experiences (PEs) at age 12 and age 18 years

Age (years)	Psychotic experiences (PEs)	Infection burden between age 1.5 and 7.5 years (no. of infections)	Sample size (no.)	% with PEs	Odds ratio (95% CI) for PEs	
					Unadjusted	Adjusted^a^
**12**	Suspected/definite	Low (0–4)	2619	12.3	1.00 (reference)	1.00 (reference)
	Definite only			5.1	1.00 (reference)	1.00 (reference)
	Definite without attribution			4.0	1.00 (reference)	1.00 (reference)
	Suspected/definite	Medium (5–6)	1561	14.0	1.20 (1.00–1.44)	1.21 (1.01–1.46)
	Definite only			5.9	1.08 (0.82–1.42)	1.11 (0.84–1.46)
	Definite without attribution			5.1	1.09 (0.81–1.47)	1.12 (0.83–1.51)
	Suspected/definite	High (7–9)	1328	14.0	1.22 (0.99–1.51)	1.24 (1.03–1.54)
	Definite only			5.9	1.15 (0.84–1.57)	1.19 (0.87–1.63)
	Definite without attribution			4.8	1.14 (0.81–1.61)	1.18 (0.84–1.67)
	Suspected/definite	Very high (10–22)	668	17.5	1.59 (1.24–2.04)	1.60 (1.25–2.05)
	Definite only			7.0	1.39 (0.97–2.01)	1.40 (0.97–2.02)
	Definite without attribution			5.8	1.40 (0.94–2.08)	1.41 (0.95– 2.10)
**18**	Suspected/definite	Low (0–4)	1783	8.8	1.00 (reference)	1.00 (reference)
	Definite only			5.2	1.00 (reference)	1.00 (reference)
	Definite without attribution			4.6	1.00 (reference)	1.00 (reference)
	Suspected/definite	Medium (5–6)	1089	8.4	0.97 (0.75–1.26)	1.01 (0.77–1.31)
	Definite only			4.5	0.87 (0.62–1.23)	0.91 (0.65–1.29)
	Definite without attribution			4.0	0.87 (0.61–1.24)	0.92 (0.64–1.32)
	Suspected/definite	High (7–9)	912	9.5	1.09 (0.81–1.47)	1.15 (0.85–1.55)
	Definite only			4.6	0.86 (0.58–1.28)	0.91 (0.61–1.36)
	Definite without attribution			3.6	0.75 (0.49–1.17)	0.81 (0.52–1.27)
	Suspected/definite	Very high (10–22)	469	10.4	1.21 (0.85–1.73)	1.23 (0.86–1.76)
	Definite only			4.7	0.88 (0.53–1.44)	0.89 (0.54–1.47)
	Definite without attribution			3.8	0.80 (0.47–1.38)	0.82 (0.48–1.42)

a Adjusted for sex, birth weight, maternal social status, ethnicity and parental history of schizophrenia.

After correction for multiple testing, evidence remained for an association between very high infection burden and suspected/definite PEs at age 12 years (*p* = <0.01) (online Supplementary Table 2). Please see Table 5 for risk associated with other categories of infection burden.

### Results based on complete case-set analyses and comparisons between analytic and missing samples

Results from the complete case-set for depressive symptoms (*N* = 1133) and that for PEs (*N* = 2495) were very similar to the main results reported above (online Supplementary Tables 3–6). See online Supplementary Tables 7 and 8 for comparison of the risk set (*N* = 11 786) with the complete case-set for depressive symptoms or PEs, respectively.

Comparison between the analytic sample for depressive symptoms (*N* = 3101) and the missing sample (*N* = 8685) showed that those included in analyses were more likely to be female (*p* = <0.001), white (*p* = 0.05), have a higher number of childhood infections (*p* = <0.001), and have lower depressive symptoms at age 10 and 18 years (*p =* <0.001) (online Supplementary Table 9). The analytic sample for depressive symptoms was less likely to have a mother of manual occupational status (*p* < 0.001) or have a parent with a history of severe depression (*p* < 0.001).

The analytic sample for PEs (*N* = 4253), compared with the missing sample (*N* = 7533), was more likely to be female (*p* = <0.001), not have a mother of manual occupational status (*p* = <0.001), and have a higher number of childhood infections (*p* = <0.001) (online Supplementary Table 10).

## Discussion

Our findings suggest that common early-childhood infections, particularly a very high infection burden, are associated with (i) the risk of depressive symptoms subsequently up to the age of 17, and (ii) the risk of suspected/definite PEs subsequently at age 12. The associations of infection with depressive symptoms at age 13 and 14 years and with suspected/definite PEs at age 12 were robust and persisted after correction for multiple testing. Childhood infections were not associated with risk of depressive symptoms or PEs at age 18/19 years.

Strong links exist between child/adolescent and adult depression (Copeland et al., 2009; Dunn & Goodyer, 2006; Fombonne et al., 2001; Lewinsohn et al., 2000). Adolescents with recurrent depressive episodes are at particularly high risk of subsequent recurrent depression as adults (Lewinsohn et al., 2000). Longitudinal studies suggest that recurrence of depression may be similar in clinic and community samples (Dunn & Goodyer, 2006). PEs in childhood or adolescence may also provide a valid method for studying the development of adult psychotic disorders (Kelleher & Cannon, 2011; Murray & Jones, 2012). PEs in childhood may be transient but population-based studies suggest that PEs in the general population and those observed in psychotic disorders may exist on a continuum (van Os, Linscott, Myin-Germeys, Delespaul, & Krabbendam, 2009). Prospective birth cohort studies have reported that PEs in childhood are associated with an increased risk of psychotic disorders in adulthood (Zammit et al., 2013). Common underlying mechanisms for PEs in healthy individuals and in schizophrenia have also been reported (Howes et al., 2013). Early-life infections could increase psychosis risk by affecting neurodevelopment, consistent with the neurodevelopmental hypothesis of schizophrenia (Murray & Lewis, 1987; Weinberger, 1987). In many cases, PEs in our sample are likely to be part of normal development while for other participants these symptoms may be more pathological (De Loore et al., 2011; Rubio, Sanjuán, Flórez-Salamanca, & Cuesta, 2012). Childhood PEs are also familial and heritable and associated with multiple risk factors for schizophrenia (Khandaker, Zammit, et al., 2014; Polanczyk et al., 2010; Thomas et al., 2009; Zammit et al., 2009). PEs and depressive symptoms in children and adolescents appear to be markers of mental distress and represent useful indicators for subsequent mental health problems.

In our sample, associations observed between childhood infections and PEs/depressive symptoms in childhood and adolescence did not persist for these outcomes in early-adulthood at age 18/19 years. There could be a number of explanations for this, including that childhood infections do not have a lasting effect on the risk for adult mental health disorders. However, findings from other long-term follow-up studies argue against this. For instance, a meta-analysis of childhood infections reported that children exposed to viral infections of the CNS were at higher risk of adult psychosis (Khandaker et al., 2012). Longitudinal studies of childhood infection and subsequent non-affective psychosis found similar results (Dalman et al., 2008; Khandaker et al., 2018). Furthermore, population-based longitudinal studies suggest that childhood infections and autoimmune disease increase risk for adult schizophrenia and mood disorders in a dose-response fashion (Benros et al., 2011, 2013). Benros and colleagues reported that a history of hospitalization for infection increases the risk of mood disorders and schizophrenia by 62% and 60%, respectively (Benros et al., 2011, 2013). One explanation for the null findings could be attrition. At age 19 years, 86% of the sample with data on childhood infections were missing from follow-up for depressive symptoms and at age 18 years, 68% of the sample were missing from follow-up for PEs. Another explanation could be the choice of exposure and outcome used. For instance, previous studies used serious infections requiring hospitalization as exposure (Benros et al., 2011, 2013), and diagnosis of schizophrenia or depression as an outcome. We have used common childhood infections as exposure and depressive symptoms/PEs as outcomes. Nevertheless, we are still able to show that relatively common childhood infections are associated with risk for depressive symptoms and PEs in adolescence. The sample size for outcomes in early-adulthood was relatively small and there was attrition over time, a common issue for prospective studies. In future, studies with larger sample sizes for cases are required.

It is possible that childhood infections contribute indirectly via inflammatory mechanisms to the risk of mental health disorders. Inflammatory responses to infection, such as elevated cytokine levels and fever, may represent the mechanism through which risk for mental disorders is increased (Flinkkilä, Keski-Rahkonen, Marttunen, & Raevuori, 2016). ‘Sickness behaviour’ is common in infection and is present in some cases of depression. Sickness behaviour is triggered by proinflammatory cytokines in response to infectious agents and includes symptoms such as fatigue, anhedonia, concentration difficulties, social withdrawal, and appetite changes (Dantzer, 2009; Dantzer et al., 2008). Along with sickness behaviour, proinflammatory cytokines may induce depression in physically ill individuals with no history of mental health disorders. Depression may be therefore a maladaptive form of cytokine-induced sickness in some individuals (Dantzer, 2009; Dantzer et al., 2008). In addition, infections in early-life have been associated with adult schizophrenia via inflammation (Al-Haddad et al., 2019; Brown et al., 2000, 2001, 2005; Brown, Begg, et al., 2004; Brown, Deicken, et al., 2009; Brown, Hooton, et al., 2004; Brown, Vinogradov, et al., 2009; Buka, Cannon, Torrey, & Yolken, 2008; Buka, Tsuang, Torrey, Klebanoff, Bernstein, et al., 2001; Buka, Tsuang, Torrey, Klebanoff, Wagner, et al., 2001; Khandaker et al., 2018; 2013; Mortensen et al., 2007, 2010; Murphy et al., 2017; Zammit et al., 2004). Infection-related inflammation may influence neurodevelopment resulting in heightened risk for schizophrenia (Benros, Mortensen, & Eaton, 2012). Inflammation represents a compelling link between infection and mental health disorders and requires further investigation.

The interaction of multiple genetic and environmental factors is likely to contribute to risk for psychosis or depression. Genetic susceptibility plays an important role in the risk of mental health disorders, but there is evidence to suggest that a significant proportion of cases may be preventable through modification of the environment (Benros, Eaton, & Mortensen, 2014; Uher, 2014). We attempted to explore the effects of environmental factors by controlling for maternal social status and birth weight. Evidence for the association between infection and PEs/depressive symptoms attenuated but did not disappear, suggesting that these environmental factors partly explain these associations. Some genetic and environmental risk factors for psychosis and depression may be shared (Uher, 2014). Childhood maltreatment, social disadvantage, and minority status are independently associated with both psychosis and depression (Uher, 2014). By recognizing infection and other environmental factors that are associated with depression and psychosis, we may be able to identify at-risk individuals early and prevent the onset of symptoms.

The strengths of this study include longitudinal design and repeated measures of both depressive symptoms and PEs. A limitation is the method for collection of childhood infection data. The questionnaire containing questions on infections were completed by primary caregiver throughout childhood with a short period of recall (past 12 months), thus minimizing, though not completely eliminating, the risk of erroneous recall. The questions on infections also require parents to have a good understanding of infectious diseases and may be vulnerable to misreporting. For example, 8.2% of parents reported that their child had no infections during childhood; a somewhat unlikely scenario. Another limitation is attrition; the number of completed mental health assessments decreases over time. This may be indicative of selective attrition of mental health cases since mental health problems have been associated with non-response and attrition (Dupuis, Strippoli, Gholam-Rezaee, Preisig, & Vandeleur, 2019). Attrition and subsequent smaller sample size could result in underestimation of the true effect of infection on mental health outcomes. Another possible explanation for the lack of association with PEs/depressive symptoms in early-adulthood could be that the relative contribution of infection to mental health becomes negligible over time due to neuroplasticity and brain development. Finally, statistical power was an issue in some of our PE analyses, resulting in wide CI.

Childhood infections are inevitable, but the adverse outcomes of such physical health problems may go beyond physical health later in life. Here we present evidence that childhood infections, particularly a very high infection burden, can negatively impact mental health well into adolescence. Future work is needed (1) to replicate the observed associations between common childhood infections and mental health outcomes during adolescence; (2) to examine whether common childhood infections have an effect on depressive symptoms and PEs in adulthood; and (3) to elucidate potential mechanisms for these associations.

## Supplementary material

For supplementary material accompanying this paper visit https://doi.org/10.1017/S0033291720004080. click here to view supplementary material

## References

[ref1] Abel, K. M., Wicks, S., Susser, E. S., Dalman, C., Pedersen, M. G., Mortensen, P. B., & Webb, R. T. (2010). Birth weight, schizophrenia, and adult mental disorder: Is risk confined to the smallest babies? Archives of General Psychiatry, 67(9), 923–930. doi: 10.1001/archgenpsychiatry.2010.100. 20819986

[ref2] Al-Haddad, B. J. S., Jacobsson, B., Chabra, S., Modzelewska, D., Olson, E. M., Bernier, R., … Sengpiel, V. (2019). Long-term risk of neuropsychiatric disease after exposure to infection in Utero. JAMA Psychiatry, 76(6), 594–602. doi: 10.1001/jamapsychiatry.2019.0029. 30840048 PMC6551852

[ref3] Bechter, K., Reiber, H., Herzog, S., Fuchs, D., Tumani, H., & Maxeiner, H. G. (2010). Cerebrospinal fluid analysis in affective and schizophrenic spectrum disorders: Identification of subgroups with immune responses and blood–CSF barrier dysfunction. Journal of Psychiatric Research, 44(5), 321–330. doi: 10.1016/j.jpsychires.2009.08.008. 19796773

[ref4] Benros, M. E., Eaton, W. W., & Mortensen, P. B. (2014). The epidemiologic evidence linking autoimmune diseases and psychosis. Biological Psychiatry, 75(4), 300–306. doi: 10.1016/j.biopsych.2013.09.023. 24199668 PMC8797267

[ref5] Benros, M. E., Mortensen, P. B., & Eaton, W. W. (2012). Autoimmune diseases and infections as risk factors for schizophrenia. Annals of the New York Academy of Sciences, 1262(1), 56–66. doi: 10.1111/j.1749-6632.2012.06638.x. 22823436

[ref6] Benros, M. E., Nielsen, P. R., Nordentoft, M., Eaton, W. W., Dalton, S. O., & Mortensen, P. B. (2011). Autoimmune diseases and severe infections as risk factors for schizophrenia: A 30-year population-based register study. The American Journal of Psychiatry, 168(12), 1303–1310. doi: 10.1176/appi.ajp.2011.11030516. 22193673

[ref7] Benros, M. E., Waltoft, B. L., Nordentoft, M., Østergaard, S. D., Eaton, W. W., Krogh, J., & Mortensen, P. B. (2013). Autoimmune diseases and severe infections as risk factors for mood disorders: A nationwide study. JAMA Psychiatry, 70(8), 812–820. doi: 10.1001/jamapsychiatry.2013.1111. 23760347

[ref8] Boulanger, L. M., & Shatz, C. J. (2004). Immune signalling in neural development, synaptic plasticity and disease. Nature Reviews Neuroscience, 5(7), 521–531. doi: 10.1038/nrn1428. 15208694

[ref9] Boyd, A., Golding, J., Macleod, J., Lawlor, D. A., Fraser, A., Henderson, J., … Davey Smith, G. (2013). Cohort profile: The ‘children of the 90s’—the index offspring of the Avon longitudinal study of parents and children. International Journal of Epidemiology, 42(1), 111–127. doi: 10.1093/ije/dys064. 22507743 PMC3600618

[ref10] Brown, A. S., Begg, M. D., Gravenstein, S., Schaefer, C. A., Wyatt, R. J., Bresnahan, M., … Susser, E. S. (2004). Serologic evidence of prenatal influenza in the etiology of schizophrenia. Archives of General Psychiatry, 61(8), 774–780. doi: 10.1001/archpsyc.61.8.774. 15289276

[ref11] Brown, A. S., Cohen, P., Harkavy-Friedman, J., Babulas, V., Malaspina, D., Gorman, J. M., & Susser, E. S. (2001). A.E. Bennett research award. Prenatal rubella, premorbid abnormalities, and adult schizophrenia. Biological Psychiatry, 49(6), 473–486. doi: 10.1016/s0006-3223(01)01068-x. 11257233

[ref12] Brown, A. S., Deicken, R. F., Vinogradov, S., Kremen, W. S., Poole, J. H., Penner, J. D., … Schaefer, C. A. (2009). Prenatal infection and cavum septum pellucidum in adult schizophrenia. Schizophrenia Research, 108(1–3), 285–287. doi: 10.1016/j.schres.2008.11.018. 19135339 PMC2821035

[ref13] Brown, A. S., & Derkits, E. J. (2010). Prenatal infection and schizophrenia: A review of epidemiologic and translational studies. American Journal of Psychiatry, 167(3), 261–280. doi: 10.1176/appi.ajp.2009.09030361. 20123911 PMC3652286

[ref14] Brown, A. S., Hooton, J., Schaefer, C. A., Zhang, H., Petkova, E., Babulas, V., … Susser, E. S. (2004). Elevated maternal interleukin-8 levels and risk of schizophrenia in adult offspring. The American Journal of Psychiatry, 161(5), 889–895. doi: 10.1176/appi.ajp.161.5.889. 15121655

[ref15] Brown, A. S., Schaefer, C. A., Quesenberry, C. P., Liu, L., Babulas, V. P., & Susser, E. S. (2005). Maternal exposure to toxoplasmosis and risk of schizophrenia in adult offspring. The American Journal of Psychiatry, 162(4), 767–773. doi: 10.1176/appi.ajp.162.4.767. 15800151

[ref16] Brown, A. S., Schaefer, C. A., Wyatt, R. J., Goetz, R., Begg, M. D., Gorman, J. M., & Susser, E. S. (2000). Maternal exposure to respiratory infections and adult schizophrenia spectrum disorders: A prospective birth cohort study. Schizophrenia Bulletin, 26(2), 287–295. doi: 10.1093/oxfordjournals.schbul.a033453. 10885631

[ref17] Brown, A. S., Vinogradov, S., Kremen, W. S., Poole, J. H., Deicken, R. F., Penner, J. D., … Schaefer, C. A. (2009). Prenatal exposure to maternal infection and executive dysfunction in adult schizophrenia. The American Journal of Psychiatry, 166(6), 683–690. doi: 10.1176/appi.ajp.2008.08010089. 19369317 PMC2885160

[ref18] Buka, S. L., Cannon, T. D., Torrey, E. F., & Yolken, R. H., & Collaborative Study Group on the Perinatal Origins of Severe Psychiatric Disorders. (2008). Maternal exposure to herpes simplex virus and risk of psychosis among adult offspring. Biological Psychiatry, 63(8), 809–815. doi: 10.1016/j.biopsych.2007.09.022. 17981263

[ref19] Buka, S. L., Tsuang, M. T., Torrey, E. F., Klebanoff, M. A., Bernstein, D., & Yolken, R. H. (2001). Maternal infections and subsequent psychosis among offspring. Archives of General Psychiatry, 58(11), 1032–1037. doi: 10.1001/archpsyc.58.11.1032. 11695949

[ref20] Buka, S. L., Tsuang, M. T., Torrey, E. F., Klebanoff, M. A., Wagner, R. L., & Yolken, R. H. (2001). Maternal cytokine levels during pregnancy and adult psychosis. Brain, Behavior, and Immunity, 15(4), 411–420. doi: 10.1006/brbi.2001.0644. 11782107

[ref21] Cannon, M., Caspi, A., Moffitt, T. E., Harrington, H., Taylor, A., Murray, R. M., & Poulton, R. (2002). Evidence for early-childhood, pan-developmental impairment specific to schizophreniform disorder: Results from a longitudinal birth cohort. Archives of General Psychiatry, 59(5), 449–456. doi: 10.1001/archpsyc.59.5.449. 11982449

[ref22] Copeland, W. E., Shanahan, L., Costello, E. J., & Angold, A. (2009). Childhood and adolescent psychiatric disorders as predictors of young adult disorders. Archives of General Psychiatry, 66(7), 764–772. doi: 10.1001/archgenpsychiatry.2009.85. 19581568 PMC2891142

[ref23] Cordeiro, C. N., Tsimis, M., & Burd, I. (2015). Infections and brain development. Obstetrical & Gynecological Survey, 70(10), 644–655. doi: 10.1097/OGX.0000000000000236. 26490164 PMC4795171

[ref24] Dalman, C., Allebeck, P., Gunnell, D., Harrison, G., Kristensson, K., Lewis, G., … Karlsson, H. (2008). Infections in the CNS during childhood and the risk of subsequent psychotic illness: A cohort study of more than one million Swedish subjects. American Journal of Psychiatry, 165(1), 59–65. doi: 10.1176/appi.ajp.2007.07050740. 18056223

[ref25] Dantzer, R. (2009). Cytokine, sickness behavior, and depression. Immunology and Allergy Clinics of North America, 29(2), 247–264. doi: 10.1016/j.iac.2009.02.002. 19389580 PMC2740752

[ref26] Dantzer, R., O'Connor, J. C., Freund, G. G., Johnson, R. W., & Kelley, K. W. (2008). From inflammation to sickness and depression: When the immune system subjugates the brain. Nature Reviews. Neuroscience, 9(1), 46–56. doi: 10.1038/nrn2297. 18073775 PMC2919277

[ref27] De Loore, E., Gunther, N., Drukker, M., Feron, F., Sabbe, B., Deboutte, D., … Myin-Germeys, I. (2011). Persistence and outcome of auditory hallucinations in adolescence: A longitudinal general population study of 1800 individuals. Schizophrenia Research, 127(1), 252–256. doi: 10.1016/j.schres.2011.01.015. 21315559

[ref28] De Picker, L. J., Morrens, M., Chance, S. A., & Boche, D. (2017). Microglia and brain plasticity in acute psychosis and schizophrenia illness course: A meta-review. Frontiers in Psychiatry, 8, 238. doi:10.3389/fpsyt.2017.00238. 29201010 PMC5696326

[ref29] Dunn, V., & Goodyer, I. M. (2006). Longitudinal investigation into childhood-and adolescence-onset depression: Psychiatric outcome in early adulthood. The British Journal of Psychiatry, 188(3), 216–222. doi: 10.1192/bjp.188.3.216. 16507961

[ref30] Dupuis, M., Strippoli, M.-P. F., Gholam-Rezaee, M., Preisig, M., & Vandeleur, C. L. (2019). Mental disorders, attrition at follow-up, and questionnaire non-completion in epidemiologic research. Illustrations from the CoLaus|PsyCoLaus study. International Journal of Methods in Psychiatric Research, 28(4), e1805. doi: 10.1002/mpr.1805. 31568629 PMC7027429

[ref31] Eisenberger, N. I., Berkman, E. T., Inagaki, T. K., Rameson, L. T., Mashal, N. M., & Irwin, M. R. (2010). Inflammation-induced anhedonia: Endotoxin reduces ventral striatum responses to reward. Biological Psychiatry, 68(8), 748–754. doi: 10.1016/j.biopsych.2010.06.010. 20719303 PMC3025604

[ref32] Fisher, H. L., Caspi, A., Poulton, R., Meier, M. H., Houts, R., Harrington, H., … Moffitt, T. E. (2013). Specificity of childhood psychotic symptoms for predicting schizophrenia by 38 years of age: A birth cohort study. Psychological Medicine, 43(10), 2077–2086. doi: 10.1017/S0033291712003091. 23302254 PMC3758773

[ref33] Flinkkilä, E., Keski-Rahkonen, A., Marttunen, M., & Raevuori, A. (2016). Prenatal inflammation, infections and mental disorders. Psychopathology, 49(5), 317–333. doi: 10.1159/000448054. 27529630

[ref34] Fombonne, E., Wostear, G., Cooper, V., Harrington, R., & Rutter, M. (2001). The Maudsley long-term follow-up of child and adolescent depression: I. Psychiatric outcomes in adulthood. The British Journal of Psychiatry, 179(3), 210–217. doi: 10.1192/bjp.179.3.210. 11532797

[ref35] Fraser, A., Macdonald-Wallis, C., Tilling, K., Boyd, A., Golding, J., Davey Smith, G., … Lawlor, D. A. (2013). Cohort profile: The Avon longitudinal study of parents and children: ALSPAC mothers cohort. International Journal of Epidemiology, 42(1), 97–110. doi: 10.1093/ije/dys066. 22507742 PMC3600619

[ref36] Goodwin, R. D. (2011). Association between infection early in life and mental disorders among youth in the community: A cross-sectional study. BMC Public Health, 11, 878. doi: 10.1186/1471-2458-11-878. 22103993 PMC3248872

[ref37] Harrison, N. A., Brydon, L., Walker, C., Gray, M. A., Steptoe, A., & Critchley, H. D. (2009). Inflammation causes mood changes through alterations in subgenual cingulate activity and mesolimbic connectivity. Biological Psychiatry, 66(5), 407–414. doi: 10.1016/j.biopsych.2009.03.015. 19423079 PMC2885494

[ref38] Holm, S. (1979). A simple sequentially rejective multiple test procedure. Scandinavian Journal of Statistics, 6(2), 65–70. Retrieved from http://www.jstor.org/stable/4615733.

[ref39] Horwood, J., Salvi, G., Thomas, K., Duffy, L., Gunnell, D., Hollis, C., … Harrison, G. (2008). IQ And non-clinical psychotic symptoms in 12-year-olds: Results from the ALSPAC birth cohort. The British Journal of Psychiatry: The Journal of Mental Science, 193(3), 185–191. doi: 10.1192/bjp.bp.108.051904. 18757973 PMC2806573

[ref40] Howes, O. D., Shotbolt, P., Bloomfield, M., Daalman, K., Demjaha, A., Diederen, K. M. J., … Sommer, I. E. (2013). Dopaminergic function in the psychosis Spectrum: An [18F]-DOPA imaging study in healthy individuals with auditory hallucinations. Schizophrenia Bulletin, 39(4), 807–814. doi: 10.1093/schbul/sbr195. 22282457 PMC3686439

[ref41] Jamshidian, M., Jalal, S., & Jansen, C. (2014). Missmech: An R package for testing homoscedasticity, multivariate normality, and missing completely at random (MCAR). Journal of Statistical Software, 56(1), 1–31. doi: 10.18637/jss.v056.i06.

[ref42] Kelleher, I., & Cannon, M. (2011). Psychotic-like experiences in the general population: Characterizing a high-risk group for psychosis. Psychological Medicine, 41(1), 1–6. doi: 10.1017/S0033291710001005. 20624328

[ref43] Kelleher, I., Keeley, H., Corcoran, P., Lynch, F., Fitzpatrick, C., Devlin, N., … Cannon, M. (2012). Clinicopathological significance of psychotic experiences in non-psychotic young people: Evidence from four population-based studies. The British Journal of Psychiatry: The Journal of Mental Science, 201(1), 26–32. doi: 10.1192/bjp.bp.111.101543. 22500011

[ref44] Khandaker, G. M., Dalman, C., Kappelmann, N., Stochl, J., Dal, H., Kosidou, K., … Karlsson, H. (2018). Association of childhood infection with IQ and adult nonaffective psychosis in Swedish Men: A population-based longitudinal cohort and co-relative study. JAMA Psychiatry, 75(4), 356. doi: 10.1001/jamapsychiatry.2017.4491. 29450471 PMC5875340

[ref45] Khandaker, G. M., Dantzer, R., & Jones, P. B. (2017). Immunopsychiatry: Important facts. Psychological Medicine, 47(13), 2229–2237. doi: 10.1017/S0033291717000745. 28418288 PMC5817424

[ref46] Khandaker, G. M., Stochl, J., Zammit, S., Lewis, G., & Jones, P. B. (2014). Childhood Epstein-Barr virus infection and subsequent risk of psychotic experiences in adolescence: A population-based prospective serological study. Schizophrenia Research, 158(0), 19–24. doi: 10.1016/j.schres.2014.05.019. 25048425 PMC4561501

[ref47] Khandaker, G. M., Zammit, S., Lewis, G., & Jones, P. B. (2014). A population-based study of atopic disorders and inflammatory markers in childhood before psychotic experiences in adolescence. Schizophrenia Research, 152(1), 139–145. doi: 10.1016/j.schres.2013.09.021. 24268471 PMC3906534

[ref48] Khandaker, G. M., Zimbron, J., Dalman, C., Lewis, G., & Jones, P. B. (2012). Childhood infection and adult schizophrenia: A meta-analysis of population-based studies. Schizophrenia Research, 139(1–3), 161–168. doi: 10.1016/j.schres.2012.05.023. 22704639 PMC3485564

[ref49] Khandaker, G. M., Zimbron, J., Lewis, G., & Jones, P. B. (2013). Prenatal maternal infection, neurodevelopment and adult schizophrenia: A systematic review of population-based studies. Psychological Medicine, 43(2), 239–257. doi: 10.1017/S0033291712000736. 22717193 PMC3479084

[ref50] Laske, C., Zank, M., Klein, R., Stransky, E., Batra, A., Buchkremer, G., & Schott, K. (2008). Autoantibody reactivity in serum of patients with major depression, schizophrenia and healthy controls. Psychiatry Research, 158(1), 83–86. doi: 10.1016/j.psychres.2006.04.023. 18096244

[ref51] Lewinsohn, P. M., Rohde, P., Seeley, J. R., Klein, D. N., & Gotlib, I. H. (2000). Natural course of adolescent major depressive disorder in a community sample: Predictors of recurrence in young adults. The American Journal of Psychiatry, 157(10), 1584–1591. doi: 10.1176/appi.ajp.157.10.1584. 11007711

[ref52] Mackinnon, N., Zammit, S., Lewis, G., Jones, P. B., & Khandaker, G. M. (2018). Association between childhood infection, serum inflammatory markers and intelligence: Findings from a population-based prospective birth cohort study. Epidemiology and Infection, 146(2), 256–264. doi: 10.1017/S0950268817002710. 29198208 PMC5851035

[ref53] Meyer, U., Schwarz, M. J., & Müller, N. (2011). Inflammatory processes in schizophrenia: A promising neuroimmunological target for the treatment of negative/cognitive symptoms and beyond. Pharmacology & Therapeutics, 132(1), 96–110. doi: 10.1016/j.pharmthera.2011.06.003. 21704074

[ref54] Miller, A. H., Maletic, V., & Raison, C. L. (2009). Inflammation and its discontents: The role of cytokines in the pathophysiology of major depression. Biological Psychiatry, 65(9), 732–741. doi: 10.1016/j.biopsych.2008.11.029. 19150053 PMC2680424

[ref55] Mortensen, P. B., Nørgaard-Pedersen, B., Waltoft, B. L., Sørensen, T. L., Hougaard, D., Torrey, E. F., & Yolken, R. H. (2007). *Toxoplasma gondii* as a risk factor for early-onset schizophrenia: Analysis of filter paper blood samples obtained at birth. Biological Psychiatry, 61(5), 688–693. doi: 10.1016/j.biopsych.2006.05.024. 16920078

[ref56] Mortensen, P. B., Pedersen, C. B., Hougaard, D. M., Nørgaard-Petersen, B., Mors, O., Børglum, A. D., & Yolken, R. H. (2010). A Danish National Birth Cohort study of maternal HSV-2 antibodies as a risk factor for schizophrenia in their offspring. Schizophrenia Research, 122(1–3), 257–263. doi: 10.1016/j.schres.2010.06.010. 20598509

[ref57] Murphy, S. K., Fineberg, A. M., Maxwell, S. D., Alloy, L. B., Zimmermann, L., Krigbaum, N. Y., … Ellman, L. M. (2017). Maternal infection and stress during pregnancy and depressive symptoms in adolescent offspring. Psychiatry Research, 257, 102–110. doi: 10.1016/j.psychres.2017.07.025. 28750213 PMC5823248

[ref58] Murray, G. K., & Jones, P. B. (2012). Psychotic symptoms in young people without psychotic illness: Mechanisms and meaning. British Journal of Psychiatry, 201(1), 4–6. doi: 10.1192/bjp.bp.111.107789. 22753849

[ref59] Murray, R. M., & Lewis, S. W. (1987). Is schizophrenia a neurodevelopmental disorder? British Medical Journal *(*Clinical Research Ed.*)*, 295(6600), 681–682. doi: 10.1136/bmj.295.6600.681. 3117295 PMC1247717

[ref60] Nielsen, P. R., Benros, M. E., & Mortensen, P. B. (2014). Hospital contacts with infection and risk of schizophrenia: A population-based cohort study with linkage of Danish national registers. Schizophrenia Bulletin, 40(6), 1526–1532. doi: 10.1093/schbul/sbt200. 24379444 PMC4193697

[ref61] Nielsen, P. R., Laursen, T. M., & Mortensen, P. B. (2013). Association between parental hospital-treated infection and the risk of schizophrenia in adolescence and early adulthood. Schizophrenia Bulletin, 39(1), 230–237. doi: 10.1093/schbul/sbr149. 22021661 PMC3523915

[ref62] Office for National Statistics (2016). The national statistics socio-economic classification (NS-SEC). Office for National Statistics. Retrieved from https://www.ons.gov.uk/methodology/classificationsandstandards/otherclassifications/thenationalstatisticssocioeconomicclassificationnssecrebasedonsoc2010.

[ref63] Polanczyk, G., Moffitt, T. E., Arseneault, L., Cannon, M., Ambler, A., Keefe, R. S. E., … Caspi, A. (2010). Etiological and clinical features of childhood psychotic symptoms: Results from a birth cohort. Archives of General Psychiatry, 67(4), 328–338. doi: 10.1001/archgenpsychiatry.2010.14. 20368509 PMC3776482

[ref64] Poulton, R., Caspi, A., Moffitt, T. E., Cannon, M., Murray, R., & Harrington, H. (2000). Children's self-reported psychotic symptoms and adult schizophreniform disorder: A 15-year longitudinal study. Archives of General Psychiatry, 57(11), 1053–1058. doi: 10.1001/archpsyc.57.11.1053. 11074871

[ref65] Rubio, J. M., Sanjuán, J., Flórez-Salamanca, L., & Cuesta, M. J. (2012). Examining the course of hallucinatory experiences in children and adolescents: A systematic review. Schizophrenia Research, 138(2), 248–254. doi: 10.1016/j.schres.2012.03.012. 22464200

[ref66] Sharp, C., Goodyer, I. M., & Croudace, T. J. (2006). The short mood and feelings questionnaire (SMFQ): A unidimensional item response theory and categorical data factor analysis of self-report ratings from a community sample of 7-through 11-year-Old children. Journal of Abnormal Child Psychology, 34(3), 365–377. doi: 10.1007/s10802-006-9027-x. 16649000

[ref67] Thomas, K., Harrison, G., Zammit, S., Lewis, G., Horwood, J., Heron, J., … Gunnell, D. (2009). Association of measures of fetal and childhood growth with non-clinical psychotic symptoms in 12-year-olds: The ALSPAC cohort. British Journal of Psychiatry, 194(6), 521–526. doi: 10.1192/bjp.bp.108.051730. PMC2802530 19478292

[ref68] Uher, R. (2014). Gene–environment interactions in severe mental illness. Frontiers in Psychiatry, 5, 48. doi:10.3389/fpsyt.2014.00048. 24860514 PMC4030208

[ref69] van Buuren, S., & Groothuis-Oudshoorn, K. (2011). mice: Multivariate imputation by chained equations in R. Journal of Statistical Software, 45(3), 1–67. Retrieved from https://ideas.repec.org/a/jss/jstsof/v045i03.html.

[ref70] van Os, J., Linscott, R., Myin-Germeys, I., Delespaul, P., & Krabbendam, L. (2009). A systematic review and meta-analysis of the psychosis continuum: Evidence for a psychosis proneness-persistence-impairment model of psychotic disorder. Psychological Medicine, 39(2), 179–195. doi: 10.1017/S0033291708003814. 18606047

[ref71] Weinberger, D. R. (1987). Implications of normal brain development for the pathogenesis of schizophrenia. Archives of General Psychiatry, 44(7), 660–669. doi: 10.1001/archpsyc.1987.01800190080012. 3606332

[ref72] White, I. R., Royston, P., & Wood, A. M. (2011). Multiple imputation using chained equations: Issues and guidance for practice. Statistics in Medicine, 30(4), 377–399. doi: 10.1002/sim.4067. 21225900

[ref73] World Health Organisation (1994). SCAN: Schedules for clinical assessment in neuropsychiatry, version 2.0. Geneva, Switzerland: Psychiatric Publishers International/American Psychiatric Press Inc. Retrieved from https://apps.who.int/iris/handle/10665/40356.

[ref74] Zammit, S., Allebeck, P., David, A. S., Dalman, C., Hemmingsson, T., Lundberg, I., & Lewis, G. (2004). A longitudinal study of premorbid IQ score and risk of developing schizophrenia, bipolar disorder, severe depression, and other nonaffective psychoses. Archives of General Psychiatry, 61(4), 354–360. doi: 10.1001/archpsyc.61.4.354. 15066893

[ref75] Zammit, S., Kounali, D., Cannon, M., David, A. S., Gunnell, D., Heron, J., … Lewis, G. (2013). Psychotic experiences and psychotic disorders at age 18 in relation to psychotic experiences at age 12 in a Longitudinal Population-based Cohort Study. American Journal of Psychiatry, 170(7), 742–750. doi: 10.1176/appi.ajp.2013.12060768. 23639948

[ref76] Zammit, S., Odd, D., Horwood, J., Thompson, A., Thomas, K., Menezes, P., … Harrison, G. (2009). Investigating whether adverse prenatal and perinatal events are associated with non-clinical psychotic symptoms at age 12 years in the ALSPAC birth cohort. Psychological Medicine, 39(9), 1457–1467. doi: 10.1017/S0033291708005126. 19215630

