# Supplementary material for: Association between common early-childhood infection and subsequent depressive symptoms and psychotic experiences in adolescence: a population-based longitudinal birth cohort study
Source: Psychol Med. 2020 Nov 13;52(11):2166–76. doi: 10.1017/S0033291720004080 (PMC9386436; doi:10.1017/S0033291720004080)
Supplement: Supplementary file 1 [file S0033291720004080sup001.docx]

**Association between common early-childhood infection and subsequent depressive symptoms and psychotic experiences in adolescence: a population-based longitudinal birth cohort study**

Supplementary Material

| **Contents** | **Page** |
| --- | --- |
| Assessment of parental history of severe depression and schizophrenia  **Supplementary Figure 1.** Maximum available sample from the risk set with data on depressive symptoms at each follow-up from age 10 years to 19 years.  **Supplementary Figure 2.** Maximum available sample from the risk set with data on psychotic experiences at age 12 years and 18 years.  **Supplementary Table 1.** Characteristics of the imputed confounders.  **Supplementary Table 2.** Corrected *P*-value for the association between childhood infections and mental health outcomes using the Holm-Bonferroni Method.  **Supplementary Table 3.** Beta estimate (SE) for the association between 1 SD increase in childhood infections and depressive symptoms from age 10 to 19 years (N = 1133).  **Supplementary Table 4.** Odds ratio (95% CI) for the association between 1 SD increase in childhood infections and psychotic experiences (PE) at age 12 and age 18 (N = 2495).  **Supplementary Table 5.** Beta estimate (SE) for the association between childhood infection burden and depressive symptoms from age 10 to 19 years old (N = 1133).  **Supplementary Table 6.** Odds ratio (95% CI) for the association between childhood infection burden and psychotic experiences (PEs) at age 12 and age 18 (N = 2495).  **Supplementary Table 7.** Missing data: Comparison of the risk set (data for childhood infections) and the complete case-set for depressive symptoms (data for childhood infections and depressive symptoms at age 10, 13, 14, 17, 18, and 18 years).  **Supplementary Table 8.** Missing data: Comparison of the risk set (data for childhood infections) and the complete case-set for psychotic experiences (data for childhood infections and psychotic experiences at age 12 years and 18 years).  **Supplementary Table 9.** Missing data: Comparison of the analytic sample for depressive symptoms (data for childhood infections and depressive symptoms at age 19 years) and the missing sample (data for childhood infections but not depressive symptoms at age 19 years).  **Supplementary Table 10.** Missing data: Comparison of the analytic sample for psychotic experiences (data for childhood infections and psychotic experiences at age 18 years) and the missing sample (data for childhood infections but not psychotic experiences at age 18 years). | 2  3  4  5  6  9  10  11  13  15  17  19  21 |

**Assessment of parental history of severe depression and schizophrenia**

The categories from the self-reported questionnaires were: “Yes, had it recently”, “Yes in past, not now”, “No never”, or “Don’t know”. Binary variables for severe depression and schizophrenia were created for both the mother and their partner where 0 = “No never” and 1 = “Yes, had it recently” or “Yes in past, not now”. Individuals who answered “Don’t know” were excluded. Parental history of both severe depression and schizophrenia were coded as a binary variables, with “yes” being recorded if at least one parent reported to having severe depression or schizophrenia, respectively.

**Supplementary Figure 1.** Maximum available sample from the risk set with data on depressive symptoms at each follow-up from age 10 years to 19 years.


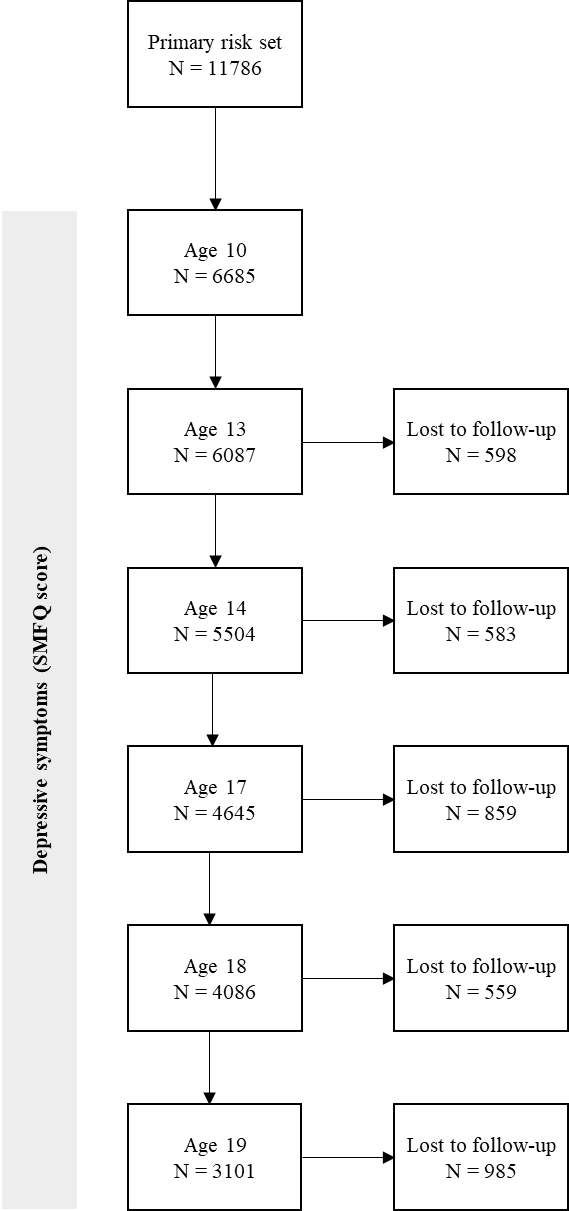


**Supplementary Figure 2.** Maximum available sample from the risk set with data on psychotic experiences at age 12 years and 18 years.


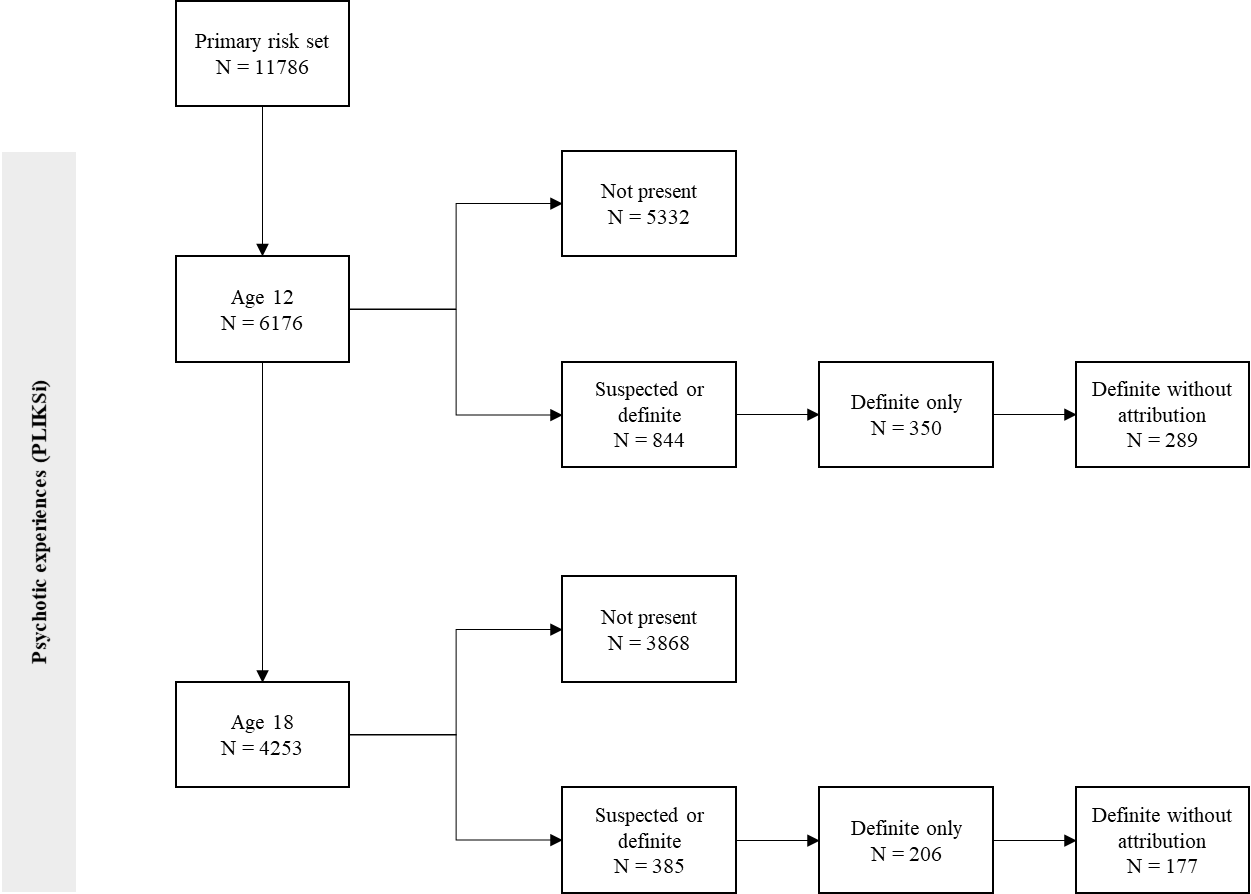


**Main analysis using imputed confounders**

| Supplementary Table 1. Characteristics of the imputed confounders. | | | | |  |  |
| --- | --- | --- | --- | --- | --- | --- |
| Confounder | Before imputation | | | After imputation | | Difference between groups (t-test p-value) |
|  | % missing data | Sample | Value | Sample | Value |  |
| Birthweight (kg) *–* *mean (SD)* | 1.5 | 11613 | 3.4 (0.5) | 11786 | 3.4 (0.5) | 0.90 |
| Ethnicity – *no.* *% white* | 6.0 | 11080 | 10828 (97.7) | 11786 | 11511 (97.7) | 0.70 |
| Maternal social status – *no. manual (*%) | 22.1 | 9183 | 1752 (19.1) | 11786 | 2621 (22.2) | <0.001 |
| Parental history of severe depression – *no. (%)* | 35.0 | 7664 | 979 (12.8) | 11786 | 1677 (14.2) | <0.001 |
| Parental history of schizophrenia *– no. (%)* | 35.0 | 7685 | 18 (0.2) | 11786 | 40 (0.3) | 0.06 |
| Total missing data | 19.9 | -- | -- | -- | -- | -- |

**Holm-Bonferroni *P*-value Correction**

| Supplementary Table 2. Corrected *P*-value for the association between childhood infections and mental health outcomes using the Holm-Bonferroni Method. | | | | |  |
| --- | --- | --- | --- | --- | --- |
| Exposure | Outcome | Age at outcome (years) | Original *P*-value * | Corrected *P*-value |  |
| Number of childhood infections | Depressive symptoms | 10 | <0.01 | 0.02 |  |
|  |  | 13 | <0.001 | <0.001 |  |
|  |  | 14 | <0.001 | 0.01 |  |
|  |  | 17 | 0.04 | 0.48 |  |
|  |  | 18 | 0.19 | 1.00 |  |
|  |  | 19 | 0.31 | 1.00 |  |
|  | *Psychotic experiences (PEs)* |  |  |  |  |
|  | Suspected/definite | 12 | <0.001 | <0.001 |  |
|  | Definite | 12 | 0.01 | 0.12 |  |
|  | Definite without attribution | 12 | 0.01 | 0.12 |  |
|  | Suspected/definite | 18 | 0.14 | 1.00 |  |
|  | Definite | 18 | 0.45 | 1.00 |  |
|  | Definite without attribution | 18 | 0.17 | 1.00 |  |
| Medium infection burden | Depressive symptoms | 10 | 0.05 | 0.62 |  |
|  |  | 13 | 0.86 | 1.00 |  |
|  |  | 14 | 0.31 | 1.00 |  |
|  |  | 17 | 0.85 | 1.00 |  |
|  |  | 18 | 0.95 | 1.00 |  |
|  |  | 19 | 0.97 | 1.00 |  |
|  | *Psychotic experiences (PEs)* |  |  |  |  |
|  | Suspected/definite | 12 | 0.04 | 0.48 |  |
|  | Definite | 12 | 0.45 | 1.00 |  |
|  | Definite without attribution | 12 | 0.47 | 1.00 |  |
|  | Suspected/definite | 18 | 0.97 | 1.00 |  |
|  | Definite | 18 | 0.61 | 1.00 |  |
|  | Definite without attribution | 18 | 0.65 | 1.00 |  |
| High infection burden | Depressive symptoms | 10 | 0.47 | 1.00 |  |
|  |  | 13 | 0.14 | 1.00 |  |
|  |  | 14 | 0.28 | 1.00 |  |
|  |  | 17 | 0.53 | 1.00 |  |
|  |  | 18 | 0.34 | 1.00 |  |
|  |  | 19 | 0.22 | 1.00 |  |
|  | *Psychotic experiences (PEs)* |  |  |  |  |
|  | Suspected/definite | 12 | 0.05 | 0.60 |  |
|  | Definite | 12 | 0.28 | 1.00 |  |
|  | Definite without attribution | 12 | 0.34 | 1.00 |  |
|  | Suspected/definite | 18 | 0.36 | 1.00 |  |
|  | Definite | 18 | 0.65 | 1.00 |  |
|  | Definite without attribution | 18 | 0.36 | 1.00 |  |
| Very high infection burden | Depressive symptoms | 10 | 0.02 | 0.24 |  |
|  |  | 13 | <0.001 | <0.001 |  |
|  |  | 14 | <0.01 | 0.02 |  |
|  |  | 17 | 0.01 | 0.12 |  |
|  |  | 18 | 0.24 | 1.00 |  |
|  |  | 19 | 0.25 | 1.00 |  |
|  | *Psychotic experiences (PEs)* |  |  |  |  |
|  | Suspected/definite | 12 | <0.001 | <0.01 |  |
|  | Definite | 12 | 0.07 | 0.84 |  |
|  | Definite without attribution | 12 | 0.09 | 1.00 |  |
|  | Suspected/definite | 18 | 0.26 | 1.00 |  |
|  | Definite | 18 | 0.66 | 1.00 |  |
|  | Definite without attribution | 18 | 0.48 | 1.00 |  |
| *The *P*-values correspond to adjusted beta estimates for depressive symptoms (presented in Table 2 and 4) or adjusted odds ratios for psychotic experiences (presented in Table 3 and 5). Beta estimates for depressive symptoms were adjusted for sex, birthweight, maternal social status, ethnicity and parental history of severe depression. Odds ratios for psychotic experiences were adjusted for sex, birthweight, maternal social status, ethnicity and parental history of schizophrenia. | | | | |  |
|  |  |  |  |  |  |

**Complete-case analysis**

| Supplementary Table 3. Beta estimate (SE) for the association between 1 SD increase in childhood infections and depressive symptoms from age 10 to 19 years (N = 1133). | | | | | |
| --- | --- | --- | --- | --- | --- |
| Age (years) | % with depression (SMFQ ≥8) | Unadjusted | | Adjusted ‡ | |
|  |  | Beta estimate (SE) for depressive symptoms | P-value | Beta estimate (SE) for depressive symptoms | P-value |
| 10 | 11.7 | 0.09 (0.10) | 0.35 | 0.08 (0.10) | 0.39 |
| 13 | 14.2 | 0.29 (0.12) | 0.01 | 0.28 (0.11) | 0.01 |
| 14 | 21.4 | 0.53 (0.14) | <0.001 | 0.51 (0.13) | <0.001 |
| 17 | 25.7 | 0.16 (0.16) | 0.32 | 0.15 (0.16) | 0.34 |
| 18 | 29.2 | 0.14 (0.15) | 0.34 | 0.14 (0.15) | 0.34 |
| 19 | 29.2 | 0.04 (0.17) | 0.82 | 0.03 (0.17) | 0.86 |
| ‡ Adjusted for sex, birthweight, maternal social status, ethnicity and parental history of severe depression. | | | | | |

| Supplementary Table 4. Odds ratio (95% CI) for the association between 1 SD increase in childhood infections and psychotic experiences (PE) at age 12 and age 18 (N = 2495). | | | | |
| --- | --- | --- | --- | --- |
| Age (years) | Psychotic experiences (PEs) | % with PEs | Odds ratio (95% CI) for PEs | |
|  |  |  | Unadjusted | Adjusted ‡ |
| 12 | Suspected/definite | 13.1 | 1.15 (1.02, 1.30) | 1.15 (1.03, 1.30) |
|  | Definite only | 5.1 | 1.13 (0.94, 1.36) | 1.13 (0.95, 1.36) |
|  | Definite without attribution | 4.4 | 1.15 (0.95, 1.39) | 1.15 (0.95, 1.40) |
| 18 | Suspected/definite | 8.1 | 1.13 (0.97, 1.31) | 1.13 (0.98, 1.31) |
|  | Definite only | 4.4 | 1.01 (0.82, 1.23) | 1.01 (0.83, 1.24) |
|  | Definite without attribution | 3.6 | 0.99 (0.80, 1.24) | 1.00 (0.80, 1.25) |
| ‡ Adjusted for sex, birthweight, maternal social status, ethnicity and parental history of schizophrenia. | | | | |

| Supplementary Table 5. Beta estimate (SE) for the association between childhood infection burden and depressive symptoms from age 10 to 19 years old  (N = 1133). | | | | | | |
| --- | --- | --- | --- | --- | --- | --- |
| Age (years) | Infection burden between age 1.5 and 7.5 years  (no. of infections) | % with depression (SMFQ ≥8) | Unadjusted | | Adjusted ‡ | |
|  |  |  | Beta estimate (SE) for depressive symptoms | P-value | Beta estimate (SE) for depressive symptoms | P-value |
| 10 | Low (0-4) | 11.8 | 0.00 (reference) |  | 0.00 (reference) |  |
|  | Medium (5-6) | 10.1 | -0.10 (0.24) | 0.68 | -0.08 (0.24) | 0.74 |
|  | High (7-9) | 11.5 | 0.02 (0.25) | 0.93 | 0.04 (0.25) | 0.86 |
|  | Very high (10-22) | 15.2 | 0.31 (0.30) | 0.31 | 0.28 (0.31) | 0.37 |
| 13 | Low (0-4) | 11.5 | 0.00 (reference) |  | 0.00 (reference) |  |
|  | Medium (5-6) | 14.2 | 0.22 (0.27) | 0.43 | 0.18 (0.27) | 0.52 |
|  | High (7-9) | 15.1 | 0.52 (0.28) | 0.07 | 0.55 (0.28) | 0.05 |
|  | Very high (10-22) | 20.0 | 0.88 (0.35) | 0.01 | 0.84 (0.35) | 0.02 |
| 14 | Low (0-4) | 17.9 | 0.00 (reference) |  | 0.00 (reference) |  |
|  | Medium (5-6) | 21.7 | 0.21 (0.32) | 0.51 | 0.12 (0.32) | 0.70 |
|  | High (7-9) | 20.8 | 0.21 (0.34) | 0.53 | 0.27 (0.33) | 0.41 |
|  | Very high (10-22) | 31.0 | 1.81 (0.42) | <0.001 | 1.68 (0.41) | <0.001 |
| 17 | Low (0-4) | 22.3 | 0.00 (reference) |  | 0.00 (reference) |  |
|  | Medium (5-6) | 26.1 | 0.63 (0.38) | 0.10 | 0.52 (0.37) | 0.17 |
|  | High (7-9) | 27.6 | 0.34 (0.40) | 0.40 | 0.49 (0.39) | 0.21 |
|  | Very high (10-22) | 30.3 | 0.65 (0.49) | 0.19 | 0.47 (0.48) | 0.33 |
| 18 | Low (0-4) | 28.9 | 0.00 (reference) |  | 0.00 (reference) |  |
|  | Medium (5-6) | 28.0 | -0.01 (0.36) | 0.98 | -0.09 (0.36) | 0.80 |
|  | High (7-9) | 27.6 | -0.13 (0.37) | 0.72 | -0.06 (0.37) | 0.88 |
|  | Very high (10-22) | 35.9 | 0.54 (0.46) | 0.25 | 0.46 (0.46) | 0.32 |
| 19 | Low (0-4) | 25.6 | 0.00 (reference) |  | 0.00 (reference) |  |
|  | Medium (5-6) | 29.6 | 0.27 (0.41) | 0.52 | 0.19 (0.41) | 0.65 |
|  | High (7-9) | 33.3 | 0.54 (0.43) | 0.21 | 0.61 (0.43) | 0.15 |
|  | Very high (10-22) | 30.3 | 0.11 (0.53) | 0.84 | <0.01 (0.53) | 1.00 |
| ‡Adjusted for sex, birthweight, maternal social status, ethnicity and parental history of severe depression. | | | | | | |

| Supplementary Table 6. Odds ratio (95% CI) for the association between childhood infection burden and psychotic experiences (PEs) at age 12 and age 18 (N = 2495). | | | | |
| --- | --- | --- | --- | --- |
| Psychotic experiences (PEs) | Infection burden between age 1.5 and 7.5 years (no. of infections) | % with PEs | Odds ratio (95% CI) for PEs | |
|  |  |  | Unadjusted | Adjusted ‡ |
| Suspected/definite age 12 | Low (0-4) | 11.8 | 1.00 (reference) | 1.00 (reference) |
| Definite only age 12 |  | 5.0 | 1.00 (reference) | 1.00 (reference) |
| Definite without attribution age 12 |  | 4.3 | 1.00 (reference) | 1.00 (reference) |
| Suspected/definite age 12 | Medium (5-6) | 12.1 | 1.03 (0.76, 1.40) | 1.04 (0.77, 1.42) |
| Definite only age 12 |  | 4.0 | 0.79 (0.48, 1.28) | 0.79 (0.49, 1.29) |
| Definite without attribution age 12 |  | 3.5 | 0.82 (0.49, 1.38) | 0.83 (0.49, 1.39) |
| Suspected/definite age 12 | High (7-9) | 15.1 | 1.33 (0.99, 1.63) | 1.35 (1.00, 1.82) |
| Definite only age 12 |  | 6.4 | 1.30 (0.84, 2.02) | 1.33 (0.86, 2.07) |
| Definite without attribution age 12 |  | 5.8 | 1.37 (0.86, 2.19) | 1.39 (0.87, 2.23) |
| Suspected/definite age 12 | Very high (10-22) | 15.5 | 1.37 (0.94, 2.00) | 1.37 (0.94, 1.99) |
| Definite only age 12 |  | 5.2 | 1.03 (0.57, 1.87) | 1.02 (0.56, 1.85) |
| Definite without attribution age 12 |  | 4.5 | 1.05 (0.55, 1.99) | 1.04 (0.55, 1.98) |
| Suspected/definite age 18 | Low (0-4) | 7.3 | 1.00 (reference) | 1.00 (reference) |
| Definite only age 18 |  | 4.5 | 1.00 (reference) | 1.00 (reference) |
| Definite without attribution age 18 |  | 3.7 | 1.00 (reference) | 1.00 (reference) |
| Suspected/definite age 18 | Medium (5-6) | 8.1 | 1.13 (0.78, 1.63) | 1.13 (0.78, 1.65) |
| Definite only age 18 |  | 4.1 | 0.92 (0.56, 1.50) | 0.93 (0.57, 1.52) |
| Definite without attribution age 18 |  | 3.5 | 0.95 (0.56, 1.61) | 0.96 (0.56, 1.63) |
| Suspected/definite age 18 | High (7-9) | 8.8 | 1.23 (0.85, 1.80) | 1.25 (0.86, 1.82) |
| Definite only age 18 |  | 4.6 | 1.02 (0.62, 1.67) | 1.04 (0.63, 1.70) |
| Definite without attribution age 18 |  | 3.6 | 0.95 (0.55, 1.65) | 0.97 (0.56, 1.68) |
| Suspected/definite age 18 | Very high (10-22) | 8.9 | 1.25 (0.78, 2.01) | 1.26 (0.78, 2.02) |
| Definite only age 18 |  | 4.5 | 1.00 (0.53, 1.88) | 1.01 (0.53, 1.91) |
| Definite without attribution age 18 |  | 3.8 | 1.01 (0.51, 2.02) | 1.02 (0.51, 2.04) |
| ‡Adjusted for sex, birthweight, maternal social status, ethnicity and parental history of schizophrenia. | | | | |

**Missing Data Comparisons**

| Supplementary Table 7. Missing data: Comparison of the risk set (data for childhood infections) and the complete case-set for depressive symptoms (data for childhood infections and depressive symptoms at age 10, 13, 14, 17, 18, and 18 years). | | | |  |
| --- | --- | --- | --- | --- |
| Characteristics | Risk set  (N = 11786) | Complete case-set for depressive symptoms  (N = 1133) | Difference between groups  (t-test p-value) | |
| Confounders |  |  |  | |
| Sex – *no. female (%)* | 5710 (48.4) | 706 (62.3) | <0.001 | |
| Ethnicity – *no. white (%)* | 11511 (97.7) | 1117 (98.6) | 0.02 | |
| Maternal social status – *no. manual (%)* | 2621 (22.2) | 120 (10.6) | <0.001 | |
| Birthweight – *mean (standard deviation (SD))* | 3.4 (0.5) | 3.4 (0.5) | 0.20 | |
| Parental history of severe depression – *no. (%)* | 1677 (14.2) | 90 (7.9) | <0.001 | |
| Number of childhood infections |  |  |  | |
| Mean (SD) | 4.6 (3.2) | 6.0 (3.0) | <0.001 | |
| Median (interquartile range) | 4 (2-6) | 6 (4-8) |  |  |
| Burden of infection – *no. (%)* |  |  |  | |
| Low (0-4 infections) | 6371 (54.1) | 391 (34.5) | <0.001 | |
| Medium (5-6 infections) | 2480 (21.0) | 318 (28.1) | <0.001 | |
| High (7-9 infections) | 1988 (16.9) | 279 (24.6) | <0.001 | |
| Very high (10-22 infections) | 947 (8.0) | 145 (12.8) | <0.001 | |
| Depressive symptoms– *mean (SD)* |  |  |  | |
| Age 10 | 4.0 (3.5) | 3.7 (3.1) | <0.001 | |
| Age 13 | 3.9 (3.8) | 3.9 (3.6) | 1.00 | |
| Age 14 | 4.9 (4.5) | 4.8 (4.3) | 0.40 | |
| Age 17 | 5.9 (5.6) | 5.5 (5.1) | 0.03 | |
| Age 18 | 6.6 (5.3) | 6.0 (4.8) | <0.001 | |
| Age 19 | 6.8 (5.9) | 6.2 (5.5) | <0.01 | |

| Supplementary Table 8. Missing data: Comparison of the risk set (data for childhood infections) and the complete case-set for psychotic experiences (data for childhood infections and psychotic experiences at age 12 years and 18 years). | | | |  |
| --- | --- | --- | --- | --- |
| Characteristics | Risk set  (N = 11786) | Complete case-set for psychotic experiences  (N = 2495) | Difference between groups  (t-test p-value) | |
| Confounders |  |  |  | |
| Sex – *no. female (%)* | 5710 (48.4) | 1372 (55.0) | <0.001 | |
| Ethnicity – *no. white (%)* | 11511 (97.7) | 2457 (98.5) | <0.01 | |
| Maternal social status – *no. manual (%)* | 2621 (22.2) | 327 (13.1) | <0.001 | |
| Birthweight – *mean (standard deviation (SD))* | 3.4 (0.5) | 3.4 (0.5) | 0.08 | |
| Parental history of schizophrenia – *no. (%)* | 40 (0.3) | 9 (0.4) | 0.90 | |
| Number of childhood infections |  |  |  | |
| Mean (SD) | 4.6 (3.2) | 5.8 (3.0) | <0.001 | |
| Median (interquartile range) | 4 (2-6) | 5 (4-8) |  |  |
| Burden of infection – *no. (%)* |  |  |  | |
| Low (0-4 infections) | 6371 (54.1) | 936 (37.5) | <0.001 | |
| Medium (5-6 infections) | 2480 (21.0) | 677 (27.1) | <0.001 | |
| High (7-9 infections) | 1988 (16.9) | 591 (23.7) | <0.001 | |
| Very high (10-22 infections) | 947 (8.0) | 291 (11.7) | <0.001 | |
| Psychotic experiences – *no. (%)* |  |  |  | |
| Suspected/definite PEs age 12 | 844 (13.7) | 326 (13.1) | 0.50 | |
| Definite PEs only age 12 | 350 (5.7) | 127 (5.1) | 0.30 | |
| Definite PEs without attribution age 12 | 289 (4.7) | 111 (4.4) | 0.60 | |
| Suspected/definite PEs age 18 | 385 (9.1) | 201 (8.1) | 0.20 | |
| Definite PEs only age 18 | 206 (4.8) | 110 (4.4) | 0.40 | |
| Definite PEs without attribution age 18 | 177 (4.2) | 91 (3.6) | 0.30 | |

| Supplementary Table 9. Missing data: Comparison of the analytic sample for depressive symptoms (data for childhood infections and depressive symptoms at age 19 years) and the missing sample (data for childhood infections but not depressive symptoms at age 19 years). | | | |  |
| --- | --- | --- | --- | --- |
| Characteristics | Analytic sample for  depressive symptoms  (N = 3101) | Missing sample for  depressive symptoms  (N = 8685) | Difference between groups  (t-test p-value) | |
| Confounders |  |  |  | |
| Sex – *no. female (%)* | 1983 (63.9) | 3727 (42.9) | <0.001 | |
| Ethnicity – *no. white (%)* | 3042 (98.1) | 8469 (97.5) | 0.05 | |
| Maternal social status – *no. manual (%)* | 456 (14.7) | 2164 (24.9) | <0.001 | |
| Birthweight – *mean (standard deviation (SD))* | 3.4 (0.5) | 3.4 (0.5) | 0.13 | |
| Parental history of severe depression – *no. (%)* | 347 (11.2) | 1330 (15.3) | <0.001 | |
| Number of childhood infections |  |  |  | |
| Mean (SD) | 5.6 (3.1) | 4.3 (3.2) | <0.001 | |
| Median (interquartile range) | 5 (3-7) | 4 (2-6) |  |  |
| Burden of infection – *no. (%)* |  |  |  | |
| Low (0-4 infections) | 1236 (40.0) | 5135 (59.1) | <0.001 | |
| Medium (5-6 infections) | 829 (26.7) | 1651 (19.0) | <0.001 | |
| High (7-9 infections) | 691 (22.3) | 1297 (14.9) | <0.001 | |
| Very high (10-22 infections) | 345 (11.1) | 602 (6.9) | <0.001 | |
| Depressive symptoms– *mean (SD)* |  |  |  | |
| Age 10 | 3.8 (3.4) | 4.2 (3.6) | <0.001 | |
| Age 13 | 4.0 (3.8) | 3.9 (3.8) | 0.10 | |
| Age 14 | 5.0 (4.5) | 4.8 (4.4) | 0.20 | |
| Age 17 | 5.8 (5.4) | 6.0 (5.8) | 0.30 | |
| Age 18 | 6.2 (5.0) | 7.0 (5.5) | <0.001 | |
| Age 19 | 6.8 (5.9) | -- | -- | |

| Supplementary Table 10. Missing data: Comparison of the analytic sample for psychotic experiences (data for childhood infections and psychotic experiences at age 18 years) and the missing sample (data for childhood infections but not psychotic experiences at age 18 years). | | | |  |
| --- | --- | --- | --- | --- |
| Characteristics | Analytic sample for psychotic experiences  (N = 4253) | Missing sample for psychotic experiences  (N = 7533) | Difference between groups  (t-test p-value) | |
| Confounders |  |  |  | |
| Sex – *no. female (%)* | 2391 (56.2) | 3319 (44.1) | <0.001 | |
| Ethnicity – *no. white (%)* | 4164 (97.9) | 7348 (97.5) | 0.23 | |
| Maternal social status – *no. manual (%)* | 708 (16.6) | 1913 (25.4) | <0.001 | |
| Birthweight – *mean (standard deviation (SD))* | 3.4 (0.5) | 3.4 (0.5) | 0.10 | |
| Parental history of schizophrenia – *no. (%)* | 17 (0.4) | 24 (0.3) | 0.51 | |
| Number of childhood infections |  |  |  | |
| Mean (SD) | 5.5 (3.1) | 4.1 (3.2) | <0.001 | |
| Median (interquartile range) | 5 (3-7) | 4 (2-6) |  |  |
| Burden of infection – *no. (%)* |  |  |  | |
| Low (0-4 infections) | 1783 (41.9) | 4588 (60.9) | <0.001 | |
| Medium (5-6 infections) | 1089 (25.6) | 1391 (18.5) | <0.001 | |
| High (7-9 infections) | 912 (21.4) | 1076 (14.3) | <0.001 | |
| Very high (10-22 infections) | 469 (11.0) | 478 (6.4) | <0.001 | |
| Psychotic experiences – *no. (%)* |  |  |  | |
| Suspected/definite PEs age 12 | 504 (13.4) | 340 (14.0) | 0.50 | |
| Definite PEs only age 12 | 203 (5.4) | 147 (6.1) | 0.30 | |
| Definite PEs without attribution age 12 | 174 (4.6) | 115 (4.8) | 0.80 | |
| Suspected/definite PEs age 18 | 385 (9.1) | -- | -- | |
| Definite PEs only age 18 | 206 (4.8) | -- | -- | |
| Definite PEs without attribution age 18 | 177 (4.2) | -- | -- | |
